# Supplementary material for: ERK-mediated NELF-A phosphorylation promotes transcription elongation of immediate-early genes by releasing promoter-proximal pausing of RNA polymerase II
Source: Nat Commun. 2022 Dec 3;13:7476. doi: 10.1038/s41467-022-35230-4 (PMC9719515; doi:10.1038/s41467-022-35230-4)
Supplement: Supplementary file 1 — Supplementary Information [file 41467_2022_35230_MOESM1_ESM.pdf]

Supplementary Information

ERK-mediated NELF-A phosphorylation promotes transcription elongation of immediate-early genes by releasing promoter-proximal pausing of RNA polymerase II

Seina Ohe[1,2], Yuji Kubota[1], Kiyoshi Yamaguchi[3], Yusuke Takagi[1], Junichiro Nashimoto[1], Hiroko Kozuka-Hata[4], Masaaki Oyama[4], Yoichi Furukawa[3], and Mutsuhiro Takekawa[1,2,4,*]

[1]Division of Cell Signaling and Molecular Medicine, Institute of Medical Science, The University of Tokyo, 4-6-1 Shirokanedai, Minato-ku, Tokyo 108-8639, Japan

[2]Department of Computational Biology and Medical Sciences, Graduate School of Frontier Sciences, The University of Tokyo, Chiba 277-8583, Japan

[3]Division of Clinical Genome Research, Institute of Medical Science, The University of Tokyo, 4-6-1 Shirokanedai, Minato-ku, Tokyo 108-8639, Japan

[4]Medical Proteomics Laboratory, Institute of Medical Science, The University of Tokyo, 4-6-1 Shirokanedai, Minato-ku, Tokyo 108-8639, Japan

*Correspondence to: takekawa@ims.u-tokyo.ac.jp

Supplementary Tables 1

Supplementary Figures 1-6

Supplementary Table 1. Oligonucleotide sequences for siRNA, shRNA, and qPCR primers.

siRNA sequences for CDK9 knockdown

Gene	Sequence
CDK9_s	5'-GGUGCUGAUGGAAAACGAGTT-3'
CDK9_as	5'-CUCGUUUUCCAUCAGCACCTT-3'

shRNA sequences for NELF-A knockdown

Gene	Sequence
shNELF-A forward	5'-gatccccGAGTGCCAGTACTTGAACAAAttcaagagaTTTGTTCAAGTACTGGCACTCttttta-3'
shNELF-A reverse	5'-agcttaaaaaGAGTGCCAGTACTTGAACAAAtctcttgaaTTTGTTCAAGTACTGGCACTCggg-3'

Primers for qRT-PCR

Gene	Sequence
FOS-F	5'-CACTCCAAGCGGAGACAGAC-3'
FOS-R	5'-GAGCTGCCAGGATGAACTCT-3'
JUNB-F	5'-CACCAAGTGCCGGAAGCGGA-3'
JUNB-R	5'-AGGGGCAGGGGACGTTCAGA-3'
FOSB-F	5'-GTGCGCCGGGAACGAATAA-3'
FOSB-R	5'-CCAACTGATCTGTCTCCGCC-3'
EGR1-F	5'-CACCTGACCGCAGAGTCTT-3'
EGR1R	5'-GCGGCCAGTATAGGTGATGG-3'
EGR2-F	5'-AGACAGGAGAGAGTAGCGAGGG-3'
EGR2-R	5'-TTTTGTCTACGGCCTTGGCG-3'
TRIB1-F	5'-AGCAGATTGTCTCCGCCGTC-3'
TRIB1-R	5'-TAAGCTGGGTTCTCTCCTCCG-3'
GADD45B-F	5'-ATTGCAACATGACGCTGGAAGAGC-3'
GADD45B-R	5'-GGATGAGCGTGAAGTGGATT-3'
GAPDH-F	5'-ATCCTGGGCTACACTGAGCA-3'
GAPDH-R	5'-GGTGGTCCAGGGGTCTTACT-3'
BTG1-F	5'-GAGCTGCTGGCAGAACATTA-3'
BTG1-R	5'-TACAACGGTAACCCGATCCC-3'
SOWAHC-F	5'-GAAAAAGGCCTCCCAGTACC-3'
SOWAHC-R	5'-GGTCCCCTCCATCTTCTAGG-3'

Primers for ChIP-qPCR (TSS position was searched on DBTSS)

Gene (position)	Sequence
FOS control-F (-2225 to -2201)	5’-GATCTAGTTGTGAATGGCAGTCATG-3’
FOS control-R (-2198 to -2175)	5’-TCAAGCTTTGAATTCCTGAGTCTG-3’
FOS TSS-F (-73 to -56)	5’-TGAGCCCGTGACGTTTAC-3’
FOS TSS-R (-5 to +13)	5’-TGCAGATGCGGTTGGAG-3’
FOS gene body (1)-F (+1070 to +1089)	5’-CGTCTCCAGTGCCAACTTCA-3’
FOS gene body (1)-R (+1105 to +1122)	5’-CCGGACTGGTCGAGATGG-3’
FOS gene body (2)-F (+1809 to +1824)	5’-GGAGGAGGGAGCTGACTGAT-3’
FOS gene body (2)-R (+1838 to +1857)	5’-ACCCACAGAGTACCTACCGC-3’
JUNB control-F (-3121 to -3101)	5’-TGCCTAGCCCACAGTAAGAGC-3’
JUNB control-R (-3034 to -3014)	5’-TGTCACGCAGACAGTGAGACT-3’
JUNB TSS-F (-3 to +15)	5’-GGCTGGGACCTTGAGAGC-3’
JUNB TSS-F (+116 to +133)	5’-GTGCGCAAAAGCCCTGTC-3’
JUNB gene body (1)-F (+707 to +726)	5’-CAAAGCCCTGGACGATCTGCACA-3’
JUNB gene body (1)-R (+732 to +726)	5’-GGGGGTGTCACGTGGTTCAT-3’
JUNB gene body (2)-F (+1317 to +1336)	5’-GGACACGCCTTCTGAACGTC-3’
JUNB gene body (2)-R (+1347 to +1366)	5’-AAGCGAGGGGGTGTCCGTAA-3’

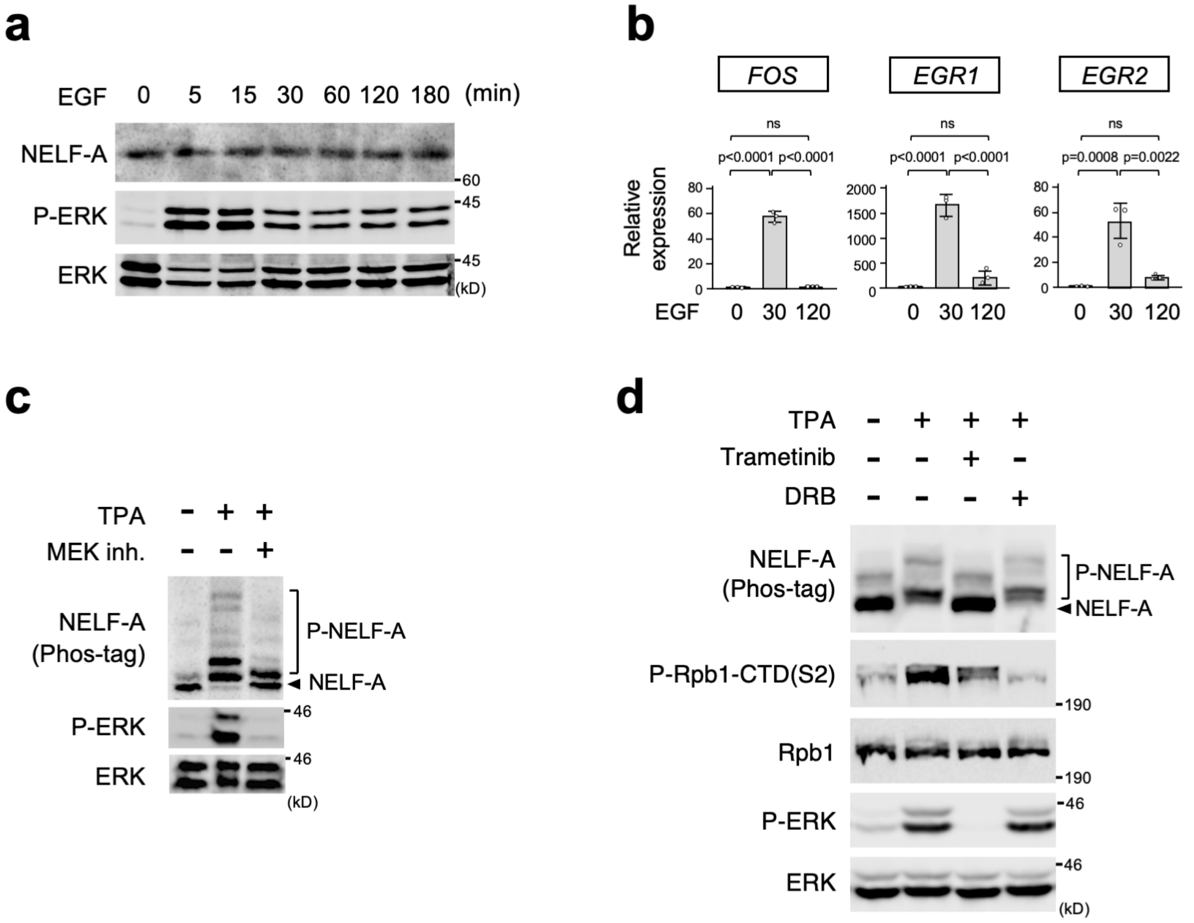

Supplementary Fig. 1 Mitogens induce NELF-A phosphorylation through the ERK pathway.

a HeLa cells were treated with EGF (20 ng/ml) for the indicated times. Cell extracts were separated on conventional SDS-PAGE and probed for endogenous NELF-A (top), phosphorylated ERK1/2 (P-ERK) (second), and ERK1/2 (bottom) by immunoblotting with the appropriate antibodies.

b HeLa cells were stimulated with EGF (20 ng/ml) for the indicated times. Total RNA was extracted and analyzed for the indicated mRNA expression using qRT-PCR. The expression levels of the IEGs are significantly reduced at 120 min after EGF, when ERK is still moderately activated but NELF-A is almost completely dephosphorylated (see Fig. 1f, left panel). All data were normalized to the level of *GAPDH* expression. Error bars, SEM (n=3). P-values were assessed using a one-way ANOVA Tukey test. ns, not significant.

c HEK293 cells were pretreated with (+) or without (-) a MEK inhibitor (trametinib, 10 μM) and stimulated with TPA (300 nM, for 45 min) as indicated. Phosphorylation status of endogenous NELF-A was analyzed as in Fig. 1f.

d Trametinib (the MEK inhibitor), but not DRB (a CDK9 inhibitor), suppresses TPA-induced NELF-A phosphorylation. HeLa cells were pretreated with trametinib (10 μM) or DRB (100 μM) and stimulated with TPA (300 nM, for 30 min) as indicated. Phosphorylation states of NELF-A and Pol II (Rpb1)-CTD(Ser2) were assessed by Phos-tag SDS-PAGE and immunoblotting, respectively. Cell lysates were probed for phosphorylated-ERK (P-ERK) and ERK by immunoblotting (lower rows). Source data are provided as a Source Data file.

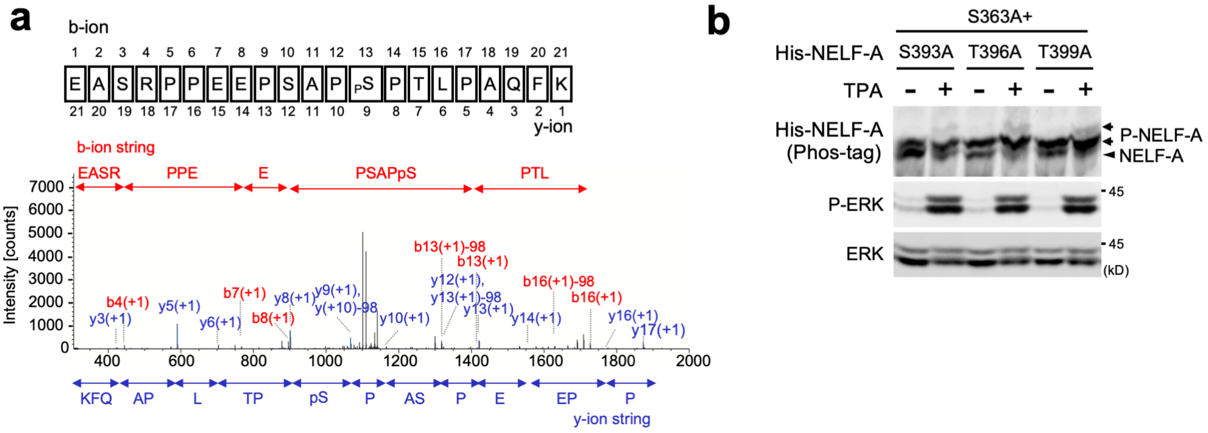

Supplementary Fig. 2 Identification of the NELF-A phosphorylation sites.

a The MS/MS spectra of the precursor ions spanning NELF-A Ser^{363}. Scheme of the predicted b- and y- ions is shown in the upper panel. The MS/MS spectra from phosphorylated NELF-A are shown in the lower panel.

b HEK293 cells were transfected with His-NELF-A with various double point mutations as indicated, and then treated with or without TPA (300 nM, for 15 min). Phosphorylation of His-NELF-A was analyzed using Phos-tag SDS-PAGE followed by immunoblotting using an anti-His antibody (upper). Cell lysates were immunoblotted for phospho-ERK1/2 and ERK1/2 (lower panels).

Source data are provided as a Source Data file.

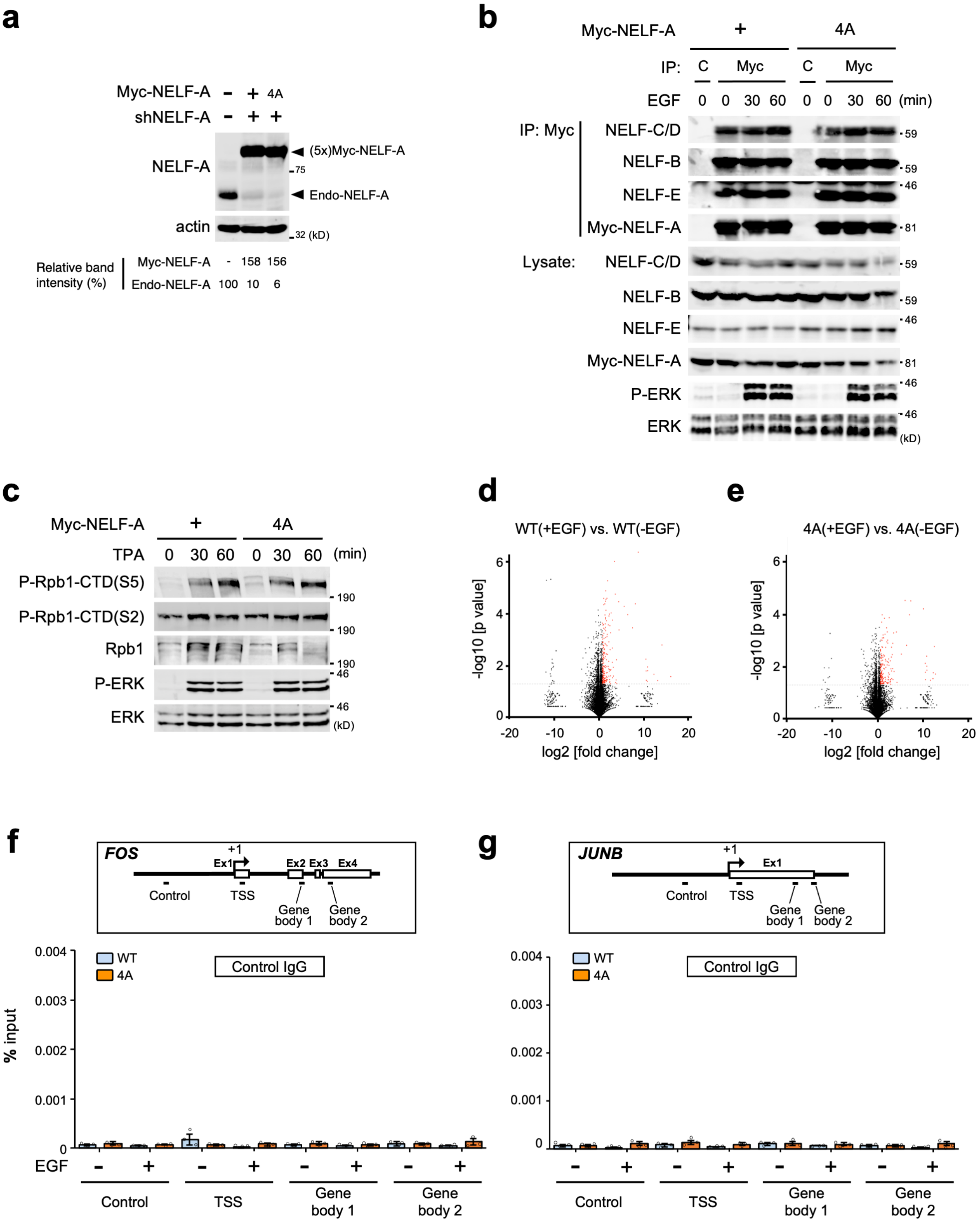

a
Myc-NELF-A
shNELF-A
NELF-A
(5x)Myc-NELF-A
Endo-NELF-A
actin
75
32 (kD)
Relative band intensity (%)
Myc-NELF-A - 158 156
Endo-NELF-A 100 10 6
b
Myc-NELF-A + 4A
IP: C Myc C Myc
EGF 0 0 30 60 0 0 30 60 (min)
IP: Myc
NELF-C/D
NELF-B
NELF-E
Myc-NELF-A
Lysate:
NELF-C/D
NELF-B
NELF-E
Myc-NELF-A
P-ERK
ERK
(kD)
c
Myc-NELF-A + 4A
TPA 0 30 60 0 30 60 (min)
P-Rpb1-CTD(S5)
P-Rpb1-CTD(S2)
Rpb1
P-ERK
ERK
(kD)
d
WT(+EGF) vs. WT(-EGF)
-log10 [p value]
log2 [fold change]
e
4A(+EGF) vs. 4A(-EGF)
-log10 [p value]
log2 [fold change]
f
FOS
+1
Ex1 Ex2 Ex3 Ex4
Control
TSS
Gene body 1
Gene body 2
WT
4A
Control IgG
% input
EGF
Control
TSS
Gene body 1
Gene body 2
g
JUNB
+1
Ex1
Control
TSS
Gene body 1
Gene body 2
WT
4A
Control IgG
Control
TSS
Gene body 1
Gene body 2

Supplementary Fig. 3 ERK-mediated NELF-A phosphorylation promotes transcriptional elongation of IEGs.

a Quantification of the expression levels of endogenous and exogenous NELF-A proteins in HEK293-WT and HEK293-4A cells. The intensity of endogenous NELF-A, Myc-NELF-A, and Myc-NELF-A(4A) bands in the western blot shown in Fig. 3a was quantified by densitometry.

b The 4A mutations do not affect the NELF complex formation. HEK293-WT and -4A cells were stimulated with EGF (20 ng/ml) for the indicated times. Myc-NELF-A (WT or 4A) was then immunoprecipitated, and coprecipitating endogenous NELF subunits (NELF-C/D, -B, and E) were detected by immunoblotting with their specific antibodies. The levels of protein expression in cell lysates are also shown in the lower rows. C, control IgG.

c HEK293-WT and -4A cells were stimulated with TPA (300 nM) for the indicated times. Cell lysates were probed for phospho-Ser5 (top) and phospho-Ser2 (second) in the C-terminal domain of Pol II (Rpb1). The total amounts of Rpb1 are also shown (third).

b,**c** ERK1/2 activation was monitored by immunoblotting using an anti-phospho-ERK antibody (P-ERK).

d,**e** Transcriptome analyses of HEK293-WT and -4A cells treated with or without EGF. The gene expression profiles of the cells were analyzed by targeted RNA sequencing using the Ion AmpliSeq transcriptome human gene expression research panel (n=3). Genes differentially expressed between before and 60 min after EGF stimulation in HEK293-WT cells (**d**) and in HEK293-4A cells (**e**) are displayed as volcano plots (x-axis: fold-change [log2], y-axis: p-value [-log10]). Red dots indicate significantly upregulated mRNA transcripts after EGF stimulation ($FC > 1.5$, $p < 0.05$).

f,**g** Control ChIP-qPCR data corresponding to the experiments shown in Figures 4b and 4d. (Upper insets) Schematic diagrams of the *FOS* (**f**) and *JUNB* (**g**) genomic loci. The boxes represent exons. The bars below the genes show the positions of amplicons used for ChIP-qPCR analysis. TSS, transcription start site. (Lower graphs) ChIP-qPCR assays were performed with control IgG and chromatin prepared from HEK293-WT or -4A cells simulated with (+) or without (-) EGF (20 ng/ml for 30 min) as indicated. Error bars, SEM (n=3).

Source data are provided as a Source Data file.

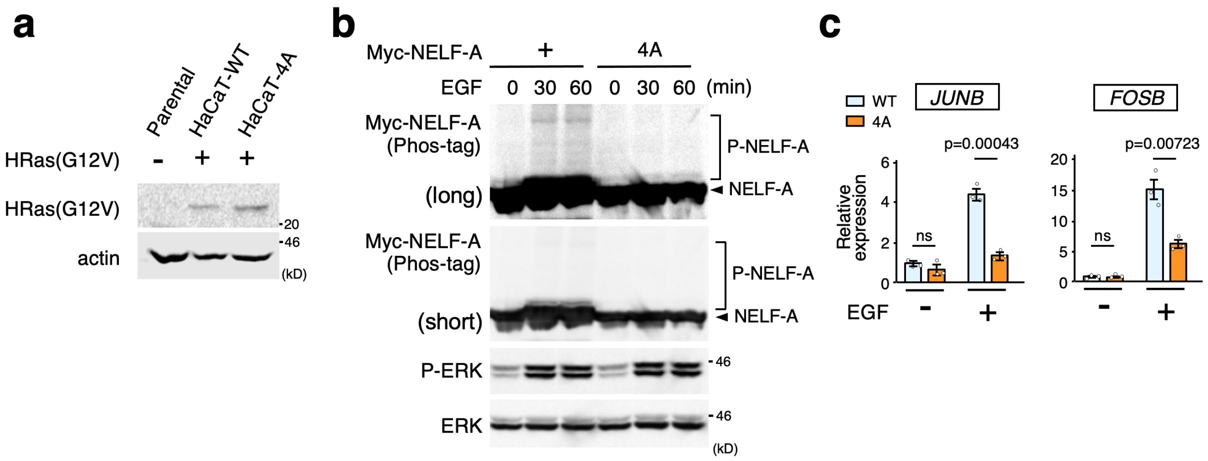

Supplementary Fig. 4 ERK-mediated NELF-A phosphorylation promotes cell growth and tumorigenesis.

a Western blot analysis of H-Ras(G12V) expression in Ras-HaCaT-WT and Ras-HaCaT-4A cells. The expression levels of an oncogenic H-Ras(G12V) mutant and actin (loading control) in Ras-HaCaT-WT and Ras-HaCaT-4A cells were analyzed by immunoblotting using an anti-Ras G12V antibody and an anti-actin antibody.

b Ras-HaCaT-WT and Ras-HaCaT-4A cells were stimulated with EGF for the indicated times. Lysates were separated on Phos-tag SDS-PAGE and immunoblotted with anti-NELF-A antibody (upper rows). Long, long exposure. Short, short exposure. P-NELF-A, phosphorylated NELF-A. Cell lysate were probed for phosphorylated ERK1/2 (P-ERK) and total ERK1/2 by immunoblotting (lower rows) as indicated.

c Ras-HaCaT-WT and Ras-HaCaT-4A cells were stimulated with or without EGF (60 ng/ml, for 60 min). Total RNA was extracted and analyzed for the indicated mRNA expression using qRT-PCR. All data were normalized to the level of *GAPDH* expression. Error bars, SEM (n=3).

Source data are provided as a Source Data file.

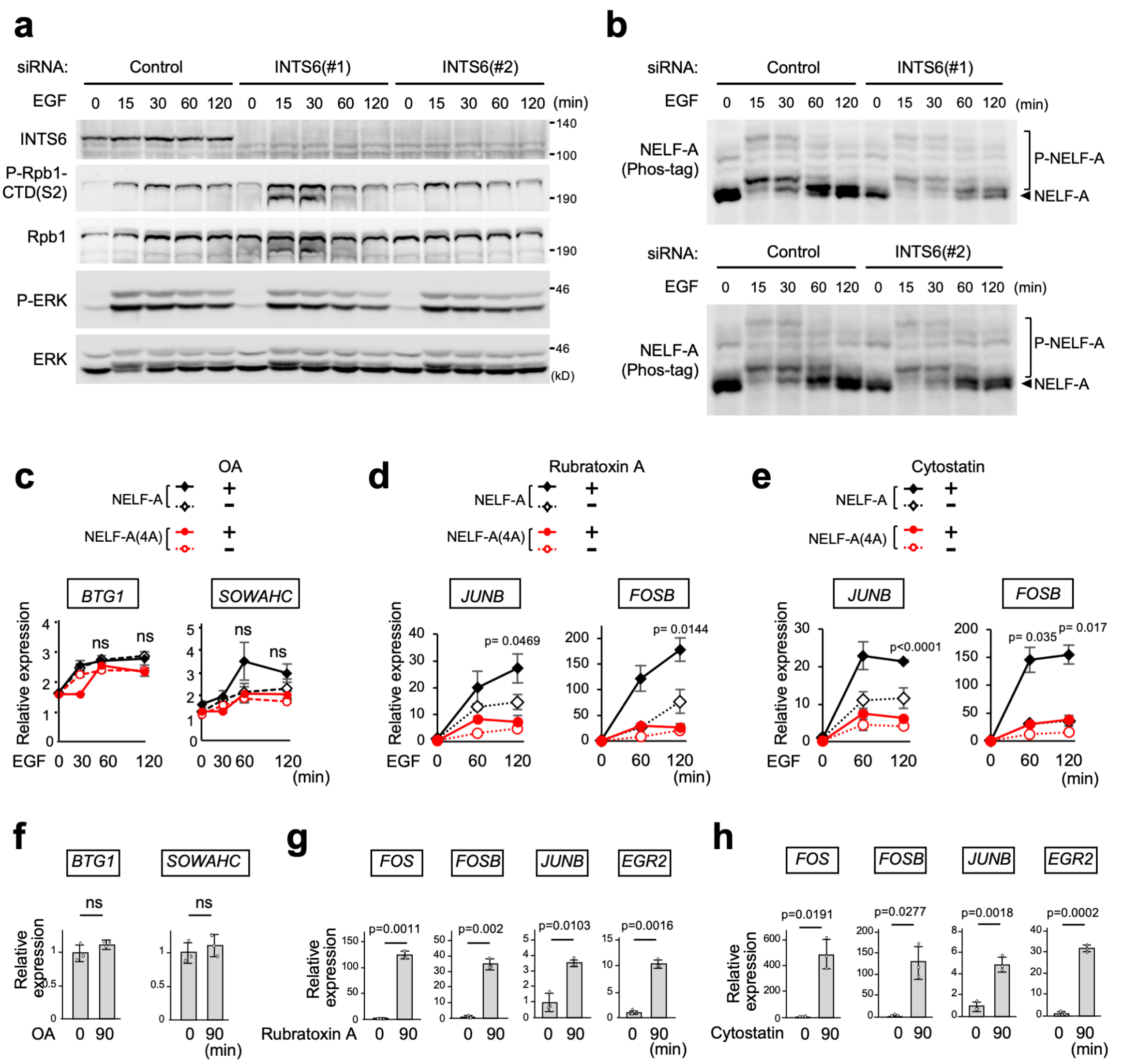

Supplementary Fig. 5 PP2A counteracts NELF-A phosphorylation.

a,**b** Inhibition of the non-canonical Integrator-PP2A complex (INTAC) suppresses dephosphorylation of Pol II-CTD(Ser2), but not that of NELF-A. HeLa cells were transfected with control siRNA or siRNAs (#1 or #2) targeting INTS6 (an essential component of INTAC). The cells were then stimulated with EGF for the indicated times. In **a**, the expression level of INTS6 and the phosphorylation states of Pol-II (Rpb1)-CTD(Ser2) and ERK were analyzed by immunoblotting. In **b**, the phosphorylation status of NELF-A was assessed using Phos-tag SDS-PAGE followed by immunoblotting with an anti-NELF-A antibody.

c,**f** Okadaic acid (OA) treatment does not significantly affect the expression levels of *BTG1* or *SOWAHC*. Since EGF stimulation upregulated the expression of these genes similarly in both HEK293-WT and -4A cells, we used them as negative controls for NELF-A phosphorylation-dependent gene regulation. In **c**, HEK293-WT (black lines) and -4A (red lines) cells were stimulated with EGF in the presence or absence of OA. In **f**, A375 cells were treated with OA for 90 min. Levels of the indicated mRNAs were quantified using qRT-PCR.

d,**e**,**g**,**h** In **d** and **e**, HEK293-WT (black lines) and -4A (red lines) cells were stimulated with EGF for the indicated times in the presence or absence of highly specific PP2A inhibitors, rubratoxin A (**d**) or cytostatin (**e**). In **g** and **h**, H1299 cells were treated with rubratoxin A (**g**) or cytostatin (**h**) for 90 min. Levels of the indicated mRNAs were quantified using qRT-PCR.

c-h, Data were normalized to *GAPDH* expression. Error bars, SEM (n=3). P-values were assessed using a two-tailed Student's t-test. Source data are provided as a Source Data file.

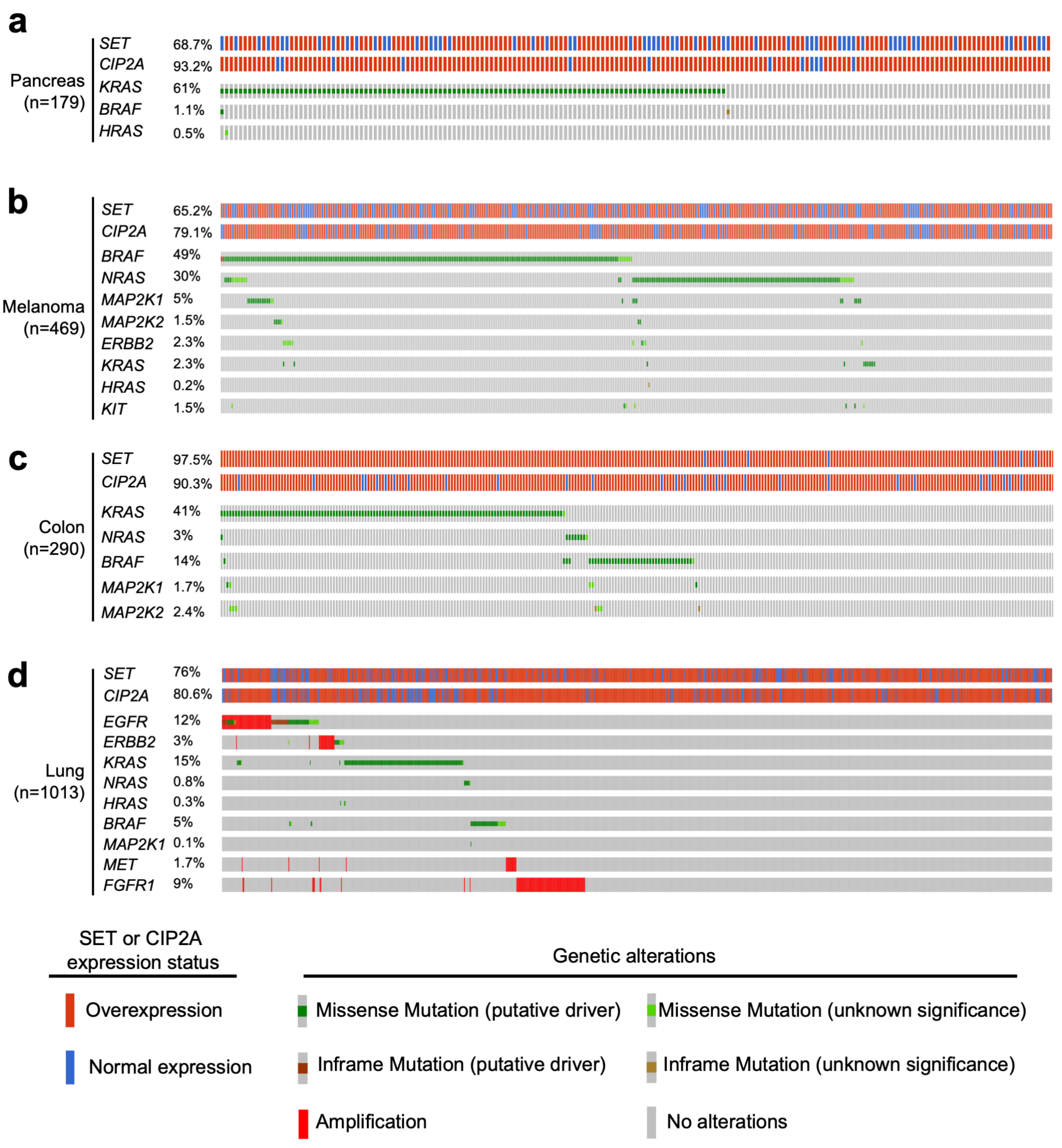

Supplementary Fig. 6 Distribution and co-occurrence of SET/CIP2A overexpression and genetic alterations in prominent ERK-activating oncogenes in various cancers.

a-**d**, OncoPrint plots showing SET/CIP2A overexpression and genetic alterations of prominent ERK-activating oncogenes in pancreas (**a**), skin melanoma (**b**), colon (**c**), and lung (**d**) cancer tissues from the TCGA database. Cancers in which SET or CIP2A expression is higher than the top 5% level of the corresponding normal tissues are defined as SET- or CIP2A-high cancers (see Fig. 8a,b). Each column represents a patient, and each row represents a gene. Numbers on the left represent the percentage of patients with alterations in a specific gene. Types of alterations are color-coded (bottom).
