# Supplementary material for: ERK-mediated NELF-A phosphorylation promotes transcription elongation of immediate-early genes by releasing promoter-proximal pausing of RNA polymerase II
Source: Nat Commun. 2022 Dec 3;13:7476. doi: 10.1038/s41467-022-35230-4 (PMC9719515; doi:10.1038/s41467-022-35230-4)
Supplement: Supplementary file 3 — Reporting Summary [file 41467_2022_35230_MOESM3_ESM.pdf]

nature portfolio

Corresponding author(s): Mutsuhiro Takekawa

Last updated by author(s): Nov 14, 2022

Reporting Summary

Nature Portfolio wishes to improve the reproducibility of the work that we publish. This form provides structure for consistency and transparency in reporting. For further information on Nature Portfolio policies, see our Editorial Policies and the Editorial Policy Checklist.

Statistics

For all statistical analyses, confirm that the following items are present in the figure legend, table legend, main text, or Methods section.

n/a	Confirmed	
☐	☒	The exact sample size (*n*) for each experimental group/condition, given as a discrete number and unit of measurement
☐	☒	A statement on whether measurements were taken from distinct samples or whether the same sample was measured repeatedly
☐	☒	The statistical test(s) used AND whether they are one- or two-sided<br>*Only common tests should be described solely by name; describe more complex techniques in the Methods section.*
☒	☐	A description of all covariates tested
☐	☒	A description of any assumptions or corrections, such as tests of normality and adjustment for multiple comparisons
☐	☒	A full description of the statistical parameters including central tendency (e.g. means) or other basic estimates (e.g. regression coefficient) AND variation (e.g. standard deviation) or associated estimates of uncertainty (e.g. confidence intervals)
☐	☒	For null hypothesis testing, the test statistic (e.g. *F*, *t*, *r*) with confidence intervals, effect sizes, degrees of freedom and *P* value noted<br>*Give P values as exact values whenever suitable.*
☒	☐	For Bayesian analysis, information on the choice of priors and Markov chain Monte Carlo settings
☒	☐	For hierarchical and complex designs, identification of the appropriate level for tests and full reporting of outcomes
☐	☒	Estimates of effect sizes (e.g. Cohen's *d*, Pearson's *r*), indicating how they were calculated

Our web collection on statistics for biologists contains articles on many of the points above.

Software and code

Policy information about availability of computer code

Data collection	Immunoblotting data were collected using the LAS-1000 plus and ImageQuant LAS-4000 (Fujifilm).<br>qRT-qPCR data were collected using Real Time PCR systems (Takara and BIO-RAD).<br>The gene expression and clinical data of cancer patients were retrieved from a combined cohort of TCGA, TARGET and GTEx samples (TCGA-TARGET-GTEx), and from the International Cancer Genome Consortium (ICGC) using the UCSC Xena Browser (https://xenabrowser.net/).
Data analysis	GraphPad Prism9 and Image J (ver. 1.53a).

For manuscripts utilizing custom algorithms or software that are central to the research but not yet described in published literature, software must be made available to editors and reviewers. We strongly encourage code deposition in a community repository (e.g. GitHub). See the Nature Portfolio guidelines for submitting code & software for further information.

Data

Policy information about availability of data

All manuscripts must include a data availability statement. This statement should provide the following information, where applicable:

- Accession codes, unique identifiers, or web links for publicly available datasets
- A description of any restrictions on data availability
- For clinical datasets or third party data, please ensure that the statement adheres to our policy

The RNA-seq data in this study were deposited in the Gene Expression Omnibus (GEO) database under accession number GSE167233 [https://

www.ncbi.nlm.nih.gov/geo/query/acc.cgi?acc=GSE167233]. The mass spectrometry proteomics data have been deposited to the ProteomeXchange Consortium via the jPOST repository with the dataset identifier PXD038094. Source data for statistical analysis of Fig. 3g, 4b, 4d, 5c, 5e-j, 7a, 7b, 8c-e, 8h, and Supplementary Fig. 1b, 3f, 3g, 4c, 5c-h are provided as a Source data file. Uncropped images of immunoblots and protein gels corresponding to those shown in Fig. 1c-i, 2b, 2c, 2e, 3a-c,5a, 5b, 5d, 6a-j, 8g, and Supplementary Fig. 1a, 1c, 1d, 2b, 3a-c, 4a, 4b, 5a, 5b are also provided in the Source data file. All other data are available in the article and its Supplementary Information. Source data are provided with this paper.

Human research participants

Policy information about studies involving human research participants and Sex and Gender in Research.

Reporting on sex and gender	n/a
Population characteristics	n/a
Recruitment	n/a
Ethics oversight	n/a

Note that full information on the approval of the study protocol must also be provided in the manuscript.

Field-specific reporting

Please select the one below that is the best fit for your research. If you are not sure, read the appropriate sections before making your selection.

[x] Life sciences [ ] Behavioural & social sciences [ ] Ecological, evolutionary & environmental sciences

For a reference copy of the document with all sections, see nature.com/documents/nr-reporting-summary-flat.pdf

Life sciences study design

All studies must disclose on these points even when the disclosure is negative.

Sample size	Sample sizes are indicated in the figure legends and/or listed within the figure panel. Statistical analyses are described in "Methods".<br>No power analysis was used for sample sizes and replicates, but these were determined based on similar studies (e.g. ref.11,and ref.17)
Data exclusions	No data was excluded from analysis.
Replication	All experiments were repeated at least three times with similar results.
Randomization	Age and sex matched nude mice (BALB-c/nu) were allocated randomly into experimental groups. All other samples were randomly allocated into experimental groups.
Blinding	Blinding was not used in this study as the individual performing analysis was also involved in collecting and labeling samples. However, all within-experiment sample groups were run at the same time so prior knowledge had no impact on data output.

Reporting for specific materials, systems and methods

We require information from authors about some types of materials, experimental systems and methods used in many studies. Here, indicate whether each material, system or method listed is relevant to your study. If you are not sure if a list item applies to your research, read the appropriate section before selecting a response.

Materials & experimental systems

n/a	Involved in the study	
[ ]	[x]	Antibodies
[ ]	[x]	Eukaryotic cell lines
[x]	[ ]	Palaeontology and archaeology
[ ]	[x]	Animals and other organisms
[x]	[ ]	Clinical data
[x]	[ ]	Dual use research of concern

Methods

n/a	Involved in the study	
[x]	[ ]	ChIP-seq
[x]	[ ]	Flow cytometry
[x]	[ ]	MRI-based neuroimaging

Antibodies

Antibodies used

The following primary antibodies were used:

anti-NELF-A G-11 (Santa Cruz Biotechnology, sc-365004) (1:3000)
anti-NELF-D C-10 (Santa Cruz Biotechnology, sc-393972) (1:2000)
anti-NELF-E F-9 (Santa Cruz Biotechnology, sc-377052) (1:2000)
anti-ERK1/2 C-9 (Santa Cruz Biotechnology, sc-514302) (1:1000)
anti-ERK1 K-23 (Santa Cruz Biotechnology, sc-94) (1:1000)
anti-Myc 9E10 (Santa Cruz Biotechnology, sc-40) (1:1000)
anti-GST B-14 (Santa Cruz Biotechnology, sc-138) (1:1000)
anti-HA F-7 (Santa Cruz Biotechnology, sc-7392) (1:1000)
anti-CyclinD1 A-12 (Santa Cruz Biotechnology, sc-8396) (1:1000)
anti-Pol II 8WG16 (Santa Cruz Biotechnology, sc-56767) (1:2000)
anti-Pol II CTD4H8 (Santa Cruz Biotechnology, sc-47701) (1:2000)
anti-CDK9 D-7 (Santa Cruz Biotechnology, sc-13130) (1:2000)
anti-Elk1 E-5 (Santa Cruz Biotechnology, sc-365876) (1:1000)
anti-Myc-Tag 9B11 (Cell Signaling Technology, 2276) (1:1000)
anti-Phospho-ERK1/2 (Cell Signaling Technology, 9101) (1:1000)
anti-Phospho-p90RSK T573 (Cell Signaling Technology, 9346) (1:1000)
anti-Phospho-Rpb1 CTD-Ser5 D9N5I (Cell Signaling Technology, 13523) (1:2000)
anti-RSK1/RSK2/RSK3 32D7 (Cell Signaling Technology, 9355) (1:1000)
anti-Phospho-Rpb1 CTD-Ser2 E1Z3G (Cell Signaling Technology, 13499) (1:1000)
anti-c-Fos 9F6 (Cell Signaling Technology, 2250) (1:1000)
anti-HA 3F10 (Roche, 11867423001) (1:1000)
anti-Flag M2 (Sigma, F1804) (1:1000)
anti β-Actin (FUJIFILM Wako, 010-27841) (1:1000)
anti-His-tag (Medical & Biological Laboratories, D291-3) (1:1000)
anti-MPM2 (anti-phospho-SP or TP) (Millipore, 05-368) (1:1000)
anti-NELF-A (Protein tech, 10456-1-AP) (1:3000)
anti-NELF-B (Protein tech, 16418-1-AP) (1:2000)
anti-GADD45B (Cloud-Clone, PAL535Hu01) (1:1000)
anti-DICE1/INST6 (Santa Cruz Biotechnology, sc-376524) (1:1000)
anti-PP2Ac (BD, 610555) (1:1000)
anti-RAS (G12V mutant) GeneTex GTX132694 (1:1000)
anti-BrdU (Monoclonal antibody from mouse, BMG 6H8 IgG1; Sigma, 5-Bromo-2′-deoxy-uridine Labeling and Detection Kit I, 11296736001)

The following secondary antibodies were used :
anti-mouse IgG-horse radish peroxidase (HRP) antibody (NA931, Cytiva) (1:5000)
anti-rabbit IgG-HRP antibody (NA934, Cytiva) (1:2500)
Alexa-Fluor 488 goat anti-mouse IgG (A-11029, Molecular Probes) (1:2000)

Validation

All antibodies used in this study were purchased from commercial vendors. Validation of antibodies used in current study is described in technical data sheets provided by manufacturers websites:

anti-NELF-A G-11 (Santa Cruz Biotechnology, sc-365004): https://www.scbt.com/p/nelf-a-antibody-g-11?requestFrom=search
anti-NELF-D C-10 (Santa Cruz Biotechnology, sc-393972) (1:2000): https://www.scbt.com/p/nelf-d-antibody-c-10?requestFrom=search
anti-NELF-E F-9 (Santa Cruz Biotechnology, sc-377052) (1:2000): https://www.scbt.com/p/nelf-e-antibody-f-9?requestFrom=search
anti-ERK1/2 C-9 (Santa Cruz Biotechnology, sc-514302) (1:1000): https://www.scbt.com/p/erk-1-2-antibody-c-9?requestFrom=search
anti-ERK1 K-23 (Santa Cruz Biotechnology, sc-94) (1:1000): https://www.scbt.com/p/erk-1-antibody-k-23?requestFrom=search
anti-Myc 9E10 (Santa Cruz Biotechnology, sc-40) (1:1000): https://www.scbt.com/p/c-myc-antibody-9e10?requestFrom=search
anti-GST B-14 (Santa Cruz Biotechnology, sc-138) (1:1000): https://www.scbt.com/p/gst-antibody-b-14?requestFrom=search
anti-HA F-7 (Santa Cruz Biotechnology, sc-7392) (1:1000): https://www.scbt.com/ja/p/ha-probe-antibody-f-7?requestFrom=search
anti-CyclinD1 A-12 (Santa Cruz Biotechnology, sc-8396) (1:1000): https://www.scbt.com/p/cyclin-d1-antibody-a-12?requestFrom=search
anti-Pol II 8WG16 (Santa Cruz Biotechnology, sc-56767) (1:2000): https://www.scbt.com/ja/p/pol-ii-antibody-8wg16?requestFrom=search
anti-Pol II CTD4H8 (Santa Cruz Biotechnology, sc-47701) (1:2000): https://www.scbt.com/p/pol-ii-antibody-ctd4h8?requestFrom=search
anti-CDK9 D-7 (Santa Cruz Biotechnology, sc-13130) (1:2000): https://www.scbt.com/ja/p/cdk9-antibody-d-7?requestFrom=search
anti-Elk1 E-5 (Santa Cruz Biotechnology, sc-365876) (1:1000): https://www.scbt.com/p/elk-1-antibody-e-5?requestFrom=search
anti-Myc-Tag 9B11 (Cell Signaling Technology, 2276) (1:1000): https://www.cellsignal.com/products/primary-antibodies/myc-tag-9b11-mouse-mab/2276
anti-Phospho-ERK1/2 (Cell Signaling Technology, 9101) (1:1000): https://www.cellsignal.com/products/primary-antibodies/phospho-p44-42-mapk-erk1-2-thr202-tyr204-antibody/9101
anti-Phospho-p90RSK T573 (Cell Signaling Technology, 9346) (1:1000): https://www.cellsignal.com/products/primary-antibodies/

phospho-p90rsk-thr573-antibody/9346
anti-Phospho-Rpb1 CTD-Ser5 D9N5I (Cell Signaling Technology, 13523) (1:2000): https://www.cellsignal.com/products/primary-antibodies/phospho-rpb1-ctd-ser5-d9n5i-rabbit-mab/13523
anti-RSK1/RSK2/RSK3 32D7 (Cell Signaling Technology, 9355) (1:1000): https://www.cellsignal.com/products/primary-antibodies/rsk1-rsk2-rsk3-32d7-rabbit-mab/9355
anti-Phospho-Rpb1 CTD-Ser2 E1Z3G (Cell Signaling Technology, 13499) (1:1000): https://www.cellsignal.com/products/primary-antibodies/phospho-rpb1-ctd-ser2-e1z3g-rabbit-mab/13499
anti-c-Fos 9F6 (Cell Signaling Technology, 2250): https://www.cellsignal.com/products/primary-antibodies/c-fos-9f6-rabbit-mab/2250
anti-HA 3F10 (Roche, 11867423001) (1:1000): https://www.sigmaaldrich.com/US/en/product/roche/roahaha?gclid=CjwKCAjwv-GUBhAzEiwASUMm4jbLh8W6TgpVxjx4EM5jcF6FhtOwfDSHFwBvzs2IKB9T7pgARtBqSBoCMAAQAvD_BwE
anti-Flag M2 (Sigma, F1804) (1:1000): https://www.sigmaaldrich.com/US/en/product/sigma/f1804
anti β-Actin (FUJIFILM Wako, 010-27841) (1:1000): https://labchem-wako.fujifilm.com/jp/product/detail/W01W0101-2784.html
anti-His-tag (Medical & Biological Laboratories, D291-3) (1:1000): https://ruo.mbl.co.jp/bio/dtl/A/index.html?pcd=D291-3
anti-MPM2 (anti-phospho-SP or TP) (Millipore, 05-368) (1:1000): https://www.merckmillipore.com/JP/ja/product/Anti-phospho-Ser-Thr-Pro-MPM-2-Antibody,MM_NF-05-368
anti-NELF-A (Protein tech, 10456-1-AP) (1:3000): https://www.ptglab.co.jp/products/NELF-A-Antibody-10456-1-AP.htm
anti-NELF-B (Protein tech, 16418-1-AP) (1:2000): https://www.ptglab.co.jp/products/NELF-B-Antibody-16418-1-AP.htm
anti-GADD45B (Cloud-Clone, PAL535Hu01) (1:1000): http://www.cloud-clone.com/products/PAL535Hu01.html
anti-DICE1/INST6 (Santa Cruz Biotechnology, sc-376524) (1:1000): https://www.scbt.com/p/dice1-antibody-h-6?requestFrom=search
anti-PP2Ac (BD, 610555) (1:1000): https://www.bdbiosciences.com/en-us/products/reagents/microscopy-imaging-reagents/immunofluorescence-reagents/purified-mouse-anti-pp2a-catalytic.610555
anti-RAS (G12V mutant) (GeneTex GTX132694) (1:1000): https://www.genetex.com/Product/Detail/RAS-G12V-Mutant-antibody/GTX132694#datasheet
anti-BrdU (BMG 6H8 IgG1; Sigma, 5-Bromo-2′-deoxy-uridine Labeling and Detection Kit I, 11296736001) : https://www.sigmaaldrich.com/US/en/product/roche/11296736001

Alexa-Fluor 488 goat anti-mouse IgG (A-21121, Molecular Probe): https://www.thermofisher.com/antibody/product/Goat-anti-Mouse-IgG-H-L-Highly-Cross-Adsorbed-Secondary-Antibody-Polyclonal/A-11029
Anti-mouse HRP antibody (NA931, Cytiva): https://www.sigmaaldrich.com/US/en/product/sigma/gena9311ml
Anti-rabbit HRP antibody (NA934, Cytiva): https://www.sigmaaldrich.com/US/en/product/sigma/gena9341ml

Eukaryotic cell lines

Policy information about cell lines and Sex and Gender in Research

Cell line source(s)	A375 (CRL-1619) and H1299 (CRL-5803) were obtained from ATCC. HEK293 (RCB1637), COS7 (RCB0539), HeLa (RCB0007), A549 (RCB3677), A431 (RCB0202), and GP2-293 (RCB2354) were obtained from RIKEN cell bank. HaCaT cells were kindly provided by Prof. Dr. N. Fusenig (German Cancer Research Center, DKFZ, Germany), who originally established this cell line.
Authentication	All cell lines used were pre-authenticated by ATCC, RIKEN cell bank, or DKFZ using STR profiling. We did not further authenticate the cell lines.
Mycoplasma contamination	All cells used in this manuscript were routinely tested for mycoplasma contamination using a PCR-based method, and verified as mycoplasma-negative.
Commonly misidentified lines (See ICLAC register)	No commonly misidentified cell lines were used in this study.

Animals and other research organisms

Policy information about studies involving animals; ARRIVE guidelines recommended for reporting animal research, and Sex and Gender in Research

Laboratory animals	Five-weeks-old female nude mice (BALB-c/nu) were purchased from Oriental Yeast (Tokyo, Japan) and bred in The Laboratory Animal Research Center (LARC) of The Institute of Medical Science, The University of Tokyo (IMSUT). Housing conditions: temperature 22 ± 2°C, humidity 55 ± 5%, light/dark cycle 12 hour/12 hour (8 am-20 pm light).
Wild animals	The study did not involve wild animals, no animals in the study were collected from the field.
Reporting on sex	The present study used female mice because they have a lower incidence of tumors compared to males, and because they are much less aggressive than males.
Field-collected samples	The study did not involve wild animals, no animals in the study were collected from the field.
Ethics oversight	The animal experiment in this study was approved by the animal experiment committee at the Institute of Medical Science, The University of Tokyo (IMSUT) (approval number: A18-47), and animal care was conducted in accordance with institutional guidelines.

Note that full information on the approval of the study protocol must also be provided in the manuscript.
